# 1,3,3-Trimethyl-5-nitro-1-phenyl­indane

**DOI:** 10.1107/S1600536810005647

**Published:** 2010-02-17

**Authors:** Xiao-Yan Ma, Di-Feng Wu, Yang Wang, Guo-Wei Gao, Jian Men

**Affiliations:** a College of Material and Chemical Engineering, Chengdu University of Technology, Chengdu 610059, People’s Republic of China; b Department of Chemistry, Sichuan University, Chengdu 610064, People’s Republic of China

## Abstract

In the title compound, C_18_H_19_NO_2_, the five-membered ring of the indane fragment adopts an envelope conformation with the unsubstituted carbon atom at the flap displaced by 0.412 (3) Å from the plane formed by the other four atoms. The nitro group forms a dihedral angle of 5.3 (2)° with the indane benzene ring while the dihedral angle between the phenyl ring and the indane benzene ring is 76.74 (9)°.

## Related literature

For general background to the synthesis, properties and applications of indane and its derivatives, see: Clark *et al.* (1998 ▶); Numata *et al.* (1976 ▶); Aliakbar *et al.* (2007 ▶). For a related structure, see: Men *et al.* (2008 ▶).
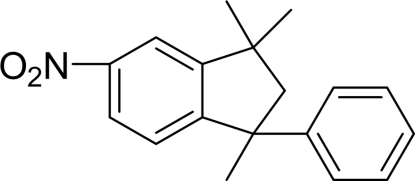


## Experimental

### 

#### Crystal data

C_18_H_19_NO_2_
*M* *_r_* = 281.34
Monoclinic, 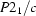

*a* = 8.306 (3) Å
*b* = 17.600 (3) Å
*c* = 12.090 (4) Å
β = 120.50 (3)°
*V* = 1522.8 (9) Å^3^
*Z* = 4
Mo *K*α radiation
μ = 0.08 mm^−1^
*T* = 292 K
0.58 × 0.48 × 0.42 mm

#### Data collection

Enraf–Nonius CAD-4 diffractometer
3123 measured reflections
2750 independent reflections
1600 reflections with *I* > 2σ(*I*)
*R*_int_ = 0.009
3 standard reflections every 200 reflections  intensity decay: 2.1%

#### Refinement

*R*[*F* ^2^ > 2σ(*F* ^2^)] = 0.057
*wR*(*F* ^2^) = 0.195
*S* = 1.12
3524 reflections
275 parameters
H-atom parameters constrained
Δρ_max_ = 0.52 e Å^−3^
Δρ_min_ = −0.40 e Å^−3^

### 

Data collection: *DIFRAC* (Gabe & White, 1993 ▶); cell refinement: *DIFRAC* data reduction: *NRCVAX* (Gabe *et al.*, 1989 ▶); program(s) used to solve structure: *SHELXS97* (Sheldrick, 2008 ▶); program(s) used to refine structure: *SHELXL97* (Sheldrick, 2008 ▶); molecular graphics: *ORTEP-3 for Windows* (Farrugia, 1997 ▶); software used to prepare material for publication: *SHELXL97*.

## Supplementary Material

Crystal structure: contains datablocks global, I. DOI: 10.1107/S1600536810005647/rz2417sup1.cif

Structure factors: contains datablocks I. DOI: 10.1107/S1600536810005647/rz2417Isup2.hkl

Additional supplementary materials:  crystallographic information; 3D view; checkCIF report

## Figures and Tables

**Fig. 1.**
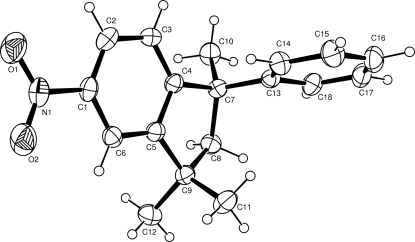
The molecular structure of the title compound, with displacement ellipsoids drawn at the 30% probability level.

## supplementary crystallographic information

### Comment

Indane has found wide industrial applications in rubber industry and as aviation fuel, lubricant, stabilizer and plasticizer (Clark *et al.*, 1998; Numata *et al.*, 1976). Indane derivatives are important intermediates for biomedical and organic synthesis. The title compound can efficiently be synthesized from 1,1,3-trimethyl-3-phenylindane by nitration (Men *et al.*, 2008; Aliakbar *et al.*, 2007), but no report on the crystal structure has been found. We report therefore herein the crystal structure of the title compound.

In the molecule of the title compound (Fig. 1), the bond lengths and angles of the phenylindane moiety are comparable with those observed in 1,1,3-trimethyl-3-phenyl-2,3-dihydro-1*H*-indane (Men *et al.*, 2008). The C4, C5, C8, C7, C9 atoms in the indane fragment are not coplanar, atom C8 deviating by 0.412 (3) Å from the plane formed by the other four atoms. The indane benzene ring (C1—C6)  and the phenyl ring (C13—18) form a dihedral angle of 76.74 (9)°. The nitro group is twisted by 5.3 (2)° with respect to the indane benzene ring. The O2—N1—C1—C2 and O1—N1—C1—C2 torsion angles are -175.0 (2)° and 4.8 (4)°, respectively.

### Experimental

1,1,3-Trimethyl-3-phenylindane (23.6 g, 0.10 mol) was dissolved in a solution of acetic anhydride (120 ml) and chloroform (30 ml) in a three-necked flask. After stirring, the mixture was cooled down to 278 K, and concentrated nitric acid (8.2 ml, 0.12 mol) was added dropwise in 30 min. Then, the mixture was stirred for 1 h at 283-289 K and poured into water (200 ml). The organic layer was washed with 10% NaOH (20 ml) and water (150 ml), then dried over anhydrous magnesium sulfate. After the solvent was removed under reduced pressure, the shallow yellow residue was recrystallized from a methanol/ethyl solution (2:1 *v*/*v*) to give a colourless solid (16.8 g, yield 59.7%, m.p. 402-404 K). Single crystals suitable for X-ray diffraction were obtained at room temperature by slow evaporation of a methanol solution over a period of several days.

### Refinement

H atoms were positioned geometrically and refined using a riding model, with C—H = 0.93–0.97 Å and with *U*_iso_(H) = 1.2*U*_eq_(C) or 1.5*U*_eq_(C) for methyl H atoms.

### Crystal data

**Table:**

C_18_H_19_NO_2_	*F*(000) = 600
*M**_r_* = 281.34	*D*_x_ = 1.227 Mg m^−^^3^
Monoclinic, *P*2_1_/*c*	Mo *K*α radiation, λ = 0.71073 Å
Hall symbol: -P 2ybc	Cell parameters from 20 reflections
*a* = 8.306 (3) Å	θ = 5.4–6.2°
*b* = 17.600 (3) Å	µ = 0.08 mm^−^^1^
*c* = 12.090 (4) Å	*T* = 292 K
β = 120.50 (3)°	Block, colourless
*V* = 1522.8 (9) Å^3^	0.58 × 0.48 × 0.42 mm
*Z* = 4	

### Data collection

**Table:**

Enraf–Nonius CAD4 diffractometer	*R*_int_ = 0.009
Radiation source: fine-focus sealed tube	θ_max_ = 25.4°, θ_min_ = 1.7°
graphite	*h* = −9→10
ω/2–θ scans	*k* = −21→0
3123 measured reflections	*l* = −8→14
2750 independent reflections	3 standard reflections every 200 reflections
1600 reflections with *I* > 2σ(*I*)	intensity decay: 2.1%

### Refinement

**Table:**

Refinement on *F*^2^	Primary atom site location: structure-invariant direct methods
Least-squares matrix: full	Secondary atom site location: difference Fourier map
*R*[*F*^2^ > 2σ(*F*^2^)] = 0.057	Hydrogen site location: inferred from neighbouring sites
*wR*(*F*^2^) = 0.195	H-atom parameters constrained
*S* = 1.12	*w* = 1/[σ^2^(*F*_o_^2^) + (0.1033*P*)^2^] where *P* = (*F*_o_^2^ + 2*F*_c_^2^)/3
3524 reflections	(Δ/σ)_max_ < 0.001
275 parameters	Δρ_max_ = 0.52 e Å^−^^3^
0 restraints	Δρ_min_ = −0.40 e Å^−^^3^

### Special details

**Table:**

Geometry. All esds (except the esd in the dihedral angle between two l.s. planes) are estimated using the full covariance matrix. The cell esds are taken into account individually in the estimation of esds in distances, angles and torsion angles; correlations between esds in cell parameters are only used when they are defined by crystal symmetry. An approximate (isotropic) treatment of cell esds is used for estimating esds involving l.s. planes.
Refinement. Refinement of *F*^2^ against ALL reflections. The weighted *R*-factor wR and goodness of fit *S* are based on *F*^2^, conventional *R*-factors *R* are based on *F*, with *F* set to zero for negative *F*^2^. The threshold expression of *F*^2^ > σ(*F*^2^) is used only for calculating *R*-factors(gt) etc. and is not relevant to the choice of reflections for refinement. *R*-factors based on *F*^2^ are statistically about twice as large as those based on *F*, and *R*- factors based on ALL data will be even larger.

### Fractional atomic coordinates and isotropic or equivalent isotropic displacement parameters (Å^2^)

**Table:**

	*x*	*y*	*z*	*U*_iso_*/*U*_eq_	
O1	0.3148 (4)	0.65100 (12)	−0.0098 (2)	0.0834 (8)	
O2	0.0977 (4)	0.56794 (15)	−0.0607 (2)	0.0874 (8)	
N1	0.2608 (4)	0.58823 (15)	0.0018 (2)	0.0591 (7)	
C1	0.3981 (4)	0.53506 (15)	0.0954 (2)	0.0462 (7)	
C2	0.5784 (4)	0.55976 (15)	0.1727 (3)	0.0547 (8)	
H2	0.6123	0.6089	0.1647	0.066*	
C3	0.7087 (4)	0.51059 (14)	0.2623 (3)	0.0510 (7)	
H3	0.8313	0.5264	0.3162	0.061*	
C4	0.6543 (3)	0.43718 (14)	0.2711 (2)	0.0414 (7)	
C5	0.4721 (4)	0.41334 (14)	0.1900 (2)	0.0412 (6)	
C6	0.3404 (4)	0.46264 (15)	0.1011 (2)	0.0470 (7)	
H6	0.2174	0.4474	0.0472	0.056*	
C7	0.7734 (4)	0.37453 (13)	0.3611 (2)	0.0433 (7)	
C8	0.6503 (4)	0.30424 (14)	0.2923 (3)	0.0498 (7)	
H8A	0.6888	0.2815	0.2363	0.060*	
H8B	0.6636	0.2666	0.3550	0.060*	
C9	0.4461 (4)	0.33044 (14)	0.2134 (3)	0.0475 (7)	
C10	0.9639 (4)	0.36996 (17)	0.3688 (3)	0.0556 (8)	
H10A	0.9449	0.3681	0.2836	0.083*	
H10B	1.0286	0.3250	0.4149	0.083*	
H10C	1.0369	0.4139	0.4126	0.083*	
C11	0.3445 (4)	0.32497 (16)	0.2899 (3)	0.0629 (8)	
H11A	0.2224	0.3471	0.2409	0.094*	
H11B	0.4147	0.3518	0.3696	0.094*	
H11C	0.3330	0.2726	0.3070	0.094*	
C12	0.3375 (5)	0.28566 (17)	0.0888 (3)	0.0691 (9)	
H12A	0.3957	0.2920	0.0382	0.104*	
H12B	0.2113	0.3040	0.0416	0.104*	
H12C	0.3370	0.2328	0.1082	0.104*	
C13	0.8050 (3)	0.38424 (14)	0.4966 (2)	0.0424 (7)	
C14	0.7377 (4)	0.44583 (15)	0.5326 (3)	0.0525 (7)	
H14	0.6684	0.4832	0.4727	0.063*	
C15	0.7731 (5)	0.45205 (18)	0.6572 (3)	0.0657 (9)	
H15	0.7272	0.4936	0.6801	0.079*	
C16	0.8742 (5)	0.39802 (18)	0.7466 (3)	0.0649 (9)	
H16	0.8993	0.4030	0.8305	0.078*	
C17	0.9385 (4)	0.33629 (17)	0.7115 (3)	0.0619 (8)	
H17	1.0052	0.2986	0.7714	0.074*	
C18	0.9053 (4)	0.32970 (15)	0.5890 (3)	0.0536 (8)	
H18	0.9512	0.2876	0.5672	0.064*	

### Atomic displacement parameters (Å^2^)

**Table:**

	*U*^11^	*U*^22^	*U*^33^	*U*^12^	*U*^13^	*U*^23^
O1	0.105 (2)	0.0546 (14)	0.0831 (18)	0.0145 (13)	0.0427 (16)	0.0192 (12)
O2	0.0538 (15)	0.114 (2)	0.0807 (18)	0.0204 (14)	0.0243 (14)	0.0379 (15)
N1	0.0687 (19)	0.0662 (17)	0.0489 (15)	0.0182 (15)	0.0348 (15)	0.0129 (13)
C1	0.0531 (18)	0.0503 (16)	0.0419 (15)	0.0122 (13)	0.0290 (14)	0.0097 (12)
C2	0.066 (2)	0.0441 (15)	0.0586 (18)	0.0014 (14)	0.0355 (17)	0.0093 (14)
C3	0.0448 (17)	0.0513 (16)	0.0500 (17)	−0.0111 (13)	0.0189 (15)	−0.0031 (13)
C4	0.0422 (16)	0.0455 (14)	0.0407 (15)	0.0016 (12)	0.0240 (14)	0.0024 (11)
C5	0.0432 (15)	0.0465 (14)	0.0385 (14)	−0.0023 (12)	0.0241 (13)	−0.0024 (12)
C6	0.0435 (16)	0.0579 (17)	0.0416 (15)	0.0000 (13)	0.0232 (13)	0.0009 (13)
C7	0.0430 (16)	0.0428 (14)	0.0445 (16)	0.0007 (11)	0.0225 (14)	0.0013 (12)
C8	0.0589 (19)	0.0421 (14)	0.0493 (17)	−0.0005 (13)	0.0281 (16)	−0.0057 (13)
C9	0.0499 (17)	0.0459 (15)	0.0461 (16)	−0.0061 (12)	0.0240 (14)	−0.0021 (12)
C10	0.0440 (17)	0.0702 (19)	0.0536 (18)	0.0084 (14)	0.0255 (15)	0.0057 (14)
C11	0.063 (2)	0.0612 (18)	0.073 (2)	−0.0036 (15)	0.0405 (18)	0.0111 (16)
C12	0.070 (2)	0.0606 (19)	0.0594 (19)	−0.0165 (16)	0.0203 (18)	−0.0095 (16)
C13	0.0376 (15)	0.0455 (14)	0.0427 (15)	−0.0034 (11)	0.0194 (13)	−0.0001 (12)
C14	0.0540 (18)	0.0528 (16)	0.0495 (16)	0.0063 (13)	0.0254 (15)	−0.0006 (13)
C15	0.071 (2)	0.074 (2)	0.0593 (19)	0.0067 (18)	0.0385 (18)	−0.0074 (17)
C16	0.070 (2)	0.086 (2)	0.0445 (17)	−0.0004 (18)	0.0338 (17)	0.0021 (17)
C17	0.064 (2)	0.070 (2)	0.0500 (18)	0.0018 (16)	0.0277 (17)	0.0146 (15)
C18	0.0608 (19)	0.0491 (16)	0.0548 (18)	0.0069 (14)	0.0323 (16)	0.0074 (14)

### Geometric parameters (Å, °)

**Table:**

O1—N1	1.227 (3)	C9—C11	1.539 (4)
O2—N1	1.223 (3)	C10—H10A	0.9600
N1—C1	1.465 (3)	C10—H10B	0.9600
C1—C2	1.373 (4)	C10—H10C	0.9600
C1—C6	1.376 (4)	C11—H11A	0.9600
C2—C3	1.380 (4)	C11—H11B	0.9600
C2—H2	0.9300	C11—H11C	0.9600
C3—C4	1.390 (3)	C12—H12A	0.9600
C3—H3	0.9300	C12—H12B	0.9600
C4—C5	1.386 (4)	C12—H12C	0.9600
C4—C7	1.512 (3)	C13—C18	1.385 (4)
C5—C6	1.382 (4)	C13—C14	1.387 (3)
C5—C9	1.522 (3)	C14—C15	1.385 (4)
C6—H6	0.9300	C14—H14	0.9300
C7—C13	1.531 (3)	C15—C16	1.363 (4)
C7—C10	1.540 (4)	C15—H15	0.9300
C7—C8	1.551 (4)	C16—C17	1.370 (4)
C8—C9	1.535 (4)	C16—H16	0.9300
C8—H8A	0.9700	C17—C18	1.366 (4)
C8—H8B	0.9700	C17—H17	0.9300
C9—C12	1.524 (4)	C18—H18	0.9300
			
O2—N1—O1	123.2 (3)	C8—C9—C11	112.2 (2)
O2—N1—C1	118.3 (3)	C7—C10—H10A	109.5
O1—N1—C1	118.5 (3)	C7—C10—H10B	109.5
C2—C1—C6	123.1 (2)	H10A—C10—H10B	109.5
C2—C1—N1	118.4 (2)	C7—C10—H10C	109.5
C6—C1—N1	118.5 (3)	H10A—C10—H10C	109.5
C1—C2—C3	119.1 (2)	H10B—C10—H10C	109.5
C1—C2—H2	120.5	C9—C11—H11A	109.5
C3—C2—H2	120.5	C9—C11—H11B	109.5
C2—C3—C4	119.1 (3)	H11A—C11—H11B	109.5
C2—C3—H3	120.4	C9—C11—H11C	109.5
C4—C3—H3	120.4	H11A—C11—H11C	109.5
C5—C4—C3	120.5 (2)	H11B—C11—H11C	109.5
C5—C4—C7	111.6 (2)	C9—C12—H12A	109.5
C3—C4—C7	127.9 (2)	C9—C12—H12B	109.5
C6—C5—C4	120.6 (2)	H12A—C12—H12B	109.5
C6—C5—C9	128.0 (2)	C9—C12—H12C	109.5
C4—C5—C9	111.5 (2)	H12A—C12—H12C	109.5
C1—C6—C5	117.6 (3)	H12B—C12—H12C	109.5
C1—C6—H6	121.2	C18—C13—C14	117.5 (2)
C5—C6—H6	121.2	C18—C13—C7	119.5 (2)
C4—C7—C13	112.52 (19)	C14—C13—C7	123.0 (2)
C4—C7—C10	111.0 (2)	C15—C14—C13	120.4 (3)
C13—C7—C10	109.2 (2)	C15—C14—H14	119.8
C4—C7—C8	100.6 (2)	C13—C14—H14	119.8
C13—C7—C8	111.82 (19)	C16—C15—C14	120.9 (3)
C10—C7—C8	111.6 (2)	C16—C15—H15	119.6
C9—C8—C7	108.3 (2)	C14—C15—H15	119.6
C9—C8—H8A	110.0	C15—C16—C17	119.2 (3)
C7—C8—H8A	110.0	C15—C16—H16	120.4
C9—C8—H8B	110.0	C17—C16—H16	120.4
C7—C8—H8B	110.0	C18—C17—C16	120.5 (3)
H8A—C8—H8B	108.4	C18—C17—H17	119.8
C5—C9—C12	112.4 (2)	C16—C17—H17	119.8
C5—C9—C8	100.8 (2)	C17—C18—C13	121.5 (2)
C12—C9—C8	111.8 (2)	C17—C18—H18	119.2
C5—C9—C11	110.2 (2)	C13—C18—H18	119.2
C12—C9—C11	109.4 (2)		
			
O2—N1—C1—C2	−175.0 (2)	C10—C7—C8—C9	−144.1 (2)
O1—N1—C1—C2	4.8 (3)	C6—C5—C9—C12	45.0 (3)
O2—N1—C1—C6	5.4 (3)	C4—C5—C9—C12	−134.4 (2)
O1—N1—C1—C6	−174.9 (2)	C6—C5—C9—C8	164.2 (2)
C6—C1—C2—C3	−1.1 (4)	C4—C5—C9—C8	−15.2 (2)
N1—C1—C2—C3	179.2 (2)	C6—C5—C9—C11	−77.2 (3)
C1—C2—C3—C4	0.6 (4)	C4—C5—C9—C11	103.4 (3)
C2—C3—C4—C5	0.7 (4)	C7—C8—C9—C5	25.7 (2)
C2—C3—C4—C7	179.7 (2)	C7—C8—C9—C12	145.3 (2)
C3—C4—C5—C6	−1.5 (3)	C7—C8—C9—C11	−91.4 (3)
C7—C4—C5—C6	179.3 (2)	C4—C7—C13—C18	177.5 (2)
C3—C4—C5—C9	178.0 (2)	C10—C7—C13—C18	−58.8 (3)
C7—C4—C5—C9	−1.2 (3)	C8—C7—C13—C18	65.2 (3)
C2—C1—C6—C5	0.3 (4)	C4—C7—C13—C14	−2.6 (3)
N1—C1—C6—C5	180.0 (2)	C10—C7—C13—C14	121.0 (3)
C4—C5—C6—C1	1.0 (3)	C8—C7—C13—C14	−115.0 (3)
C9—C5—C6—C1	−178.4 (2)	C18—C13—C14—C15	0.8 (4)
C5—C4—C7—C13	−102.2 (2)	C7—C13—C14—C15	−179.1 (3)
C3—C4—C7—C13	78.7 (3)	C13—C14—C15—C16	0.0 (5)
C5—C4—C7—C10	135.1 (2)	C14—C15—C16—C17	−1.1 (5)
C3—C4—C7—C10	−44.0 (3)	C15—C16—C17—C18	1.5 (5)
C5—C4—C7—C8	16.9 (2)	C16—C17—C18—C13	−0.6 (4)
C3—C4—C7—C8	−162.2 (2)	C14—C13—C18—C17	−0.5 (4)
C4—C7—C8—C9	−26.4 (2)	C7—C13—C18—C17	179.4 (3)
C13—C7—C8—C9	93.2 (2)		
